# Impact of a food-based dietary fat exchange model for replacing dietary saturated with unsaturated fatty acids in healthy men on plasma phospholipids fatty acid profiles and dietary patterns

**DOI:** 10.1007/s00394-022-02910-2

**Published:** 2022-06-06

**Authors:** Laury Sellem, Rona Antoni, Athanasios Koutsos, Ezgi Ozen, Gloria Wong, Hasnaa Ayyad, Michelle Weech, Matthias B. Schulze, Andreas Wernitz, Barbara A. Fielding, M. Denise Robertson, Kim G. Jackson, Bruce A. Griffin, Julie A. Lovegrove

**Affiliations:** 1 Hugh Sinclair Unit of Human Nutrition, and Institute for Cardiovascular and Metabolic Research, Department of Food and Nutritional Science, University of Reading, Whiteknights, Pepper Lane, Harry Nursten Building, Reading, RG6 6DZ UK; 2 Nutritional Sciences, Faculty of Health & Medical Sciences, University of Surrey, Guildford, GU2 7WG UK; 3 Institute of Nutritional Science, University of Potsdam, Potsdam, Germany; 4 Present Address: Human Nutrition, School of Medicine, Dentistry and Nursing, College of Medical, Veterinary and Life Sciences, University of Glasgow, New Lister Building, Glasgow Royal Infirmary, Glasgow, G31 2ER UK

**Keywords:** Dietary fat composition, Food-exchange model, Dietary compliance, Dairy biomarkers, Dietary fat replacement

## Abstract

**Purpose:**

UK guidelines recommend dietary saturated fatty acids (SFAs) should not exceed 10% total energy (%TE) for cardiovascular disease prevention, with benefits observed when SFAs are replaced with unsaturated fatty acids (UFAs). This study aimed to assess the efficacy of a dietary exchange model using commercially available foods to replace SFAs with UFAs.

**Methods:**

Healthy men (*n* = 109, age 48, SD 11 year) recruited to the Reading, Imperial, Surrey, Saturated fat Cholesterol Intervention-1 (RISSCI-1) study (ClinicalTrials.Gov n°NCT03270527) followed two sequential 4-week isoenergetic moderate-fat (34%TE) diets: high-SFA (18%TE SFAs, 16%TE UFAs) and low-SFA (10%TE SFAs, 24%TE UFAs). Dietary intakes were assessed using 4-day weighed diet diaries. Nutrient intakes were analysed using paired *t*-tests, fasting plasma phospholipid fatty acid (PL-FA) profiles and dietary patterns were analysed using orthogonal partial least square discriminant analyses.

**Results:**

Participants exchanged 10.2%TE (SD 4.1) SFAs for 9.7%TE (SD 3.9) UFAs between the high and low-SFA diets, reaching target intakes with minimal effect on other nutrients or energy intakes. Analyses of dietary patterns confirmed successful incorporation of recommended foods from commercially available sources (e.g. dairy products, snacks, oils, and fats), without affecting participants’ overall dietary intakes. Analyses of plasma PL-FAs indicated good compliance to the dietary intervention and foods of varying SFA content.

**Conclusions:**

RISSCI-1 dietary exchange model successfully replaced dietary SFAs with UFAs in free-living healthy men using commercially available foods, and without altering their dietary patterns. Further intervention studies are required to confirm utility and feasibility of such food-based dietary fat replacement models at a population level.

**Supplementary Information:**

The online version contains supplementary material available at 10.1007/s00394-022-02910-2.

## Introduction

A quarter of all deaths in the UK are attributed to cardiovascular diseases (CVD), which represent a major burden on public health worldwide [1]. While the aetiology of CVD is multifactorial, elevated circulating low-density lipoprotein cholesterol (LDL-C) has been established as a causal risk factor for the development of atherosclerosis [2]. Evidence from epidemiological prospective cohort studies, strictly controlled metabolic ward studies, and randomised controlled trials supports consistent associations between a high consumption of dietary saturated fatty acids (SFAs) and elevated serum LDL-C [3–6]. This evidence has formed the basis of public health guidelines in the UK, which since 1983, have recommended dietary SFAs should not exceed 10% of total energy (%TE) intake in adults [7, 8].

To study the impact of reducing dietary SFAs on health, many previous dietary interventions replaced SFAs with unsaturated fatty acids (UFAs) i.e. mono (MUFAs) or polyunsaturated fatty acids (PUFAs) [9]. However, these studies often used dietary fats manufactured specifically for the purpose of the intervention, which limited the translation and applicability of the findings to non-experimental, free-living people settings [10–13]. This limitation raises the importance of developing interventions based on commercially available whole-foods to improve the practicability of reducing dietary SFAs and adherence to dietary guidelines, while minimising the impact on other dietary components. In particular, since about a third of dietary SFAs is consumed from dairy foods and fat spreads in UK adults aged 19–64 years [14], the replacement of full-fat dairy and butter for lower fat or plant-based alternatives has been proposed as a food-based strategy to help reduce dietary SFAs in this group [15].

In parallel with developing food-based interventions, the assessment of dietary compliance beyond traditional approaches using diet diaries, or food-frequency questionnaires linked with food composition databases, would increase understanding of the impact and feasibility of dietary intervention studies in free-living individuals. Plasma phospholipid fatty acids (PL-FAs) correlate with the short to medium-term intake of dietary fatty acid (FA) [16, 17], and as such, PL odd-chain SFAs (e.g. pentadecanoic or heptadecanoic acids) have been used as biomarkers of dairy fat consumption [18, 19]. The use of plasma PL-FA as an objective tool to assess dietary compliance may thus be particularly effective in the context of interventions that manipulate dietary fat using full-fat dairy foods. Furthermore, the analysis of dietary patterns can identify residual confounding from changes in dietary habits, which are not routinely assessed in dietary intervention studies.

The Reading, Imperial, Surrey, Saturated fat Cholesterol Intervention-1 (‘RISSCI’-1) study was based on a tailored, dietary fat-exchange model, matched to the average diet of UK adult men. The study aimed to replace dietary SFAs with UFAs using common, commercially available foods, while minimising impacts on dietary habits, and improving dietary compliance and reproducibility, with the primary outcome of measuring variability in LDL-C responses to saturated fat [20]. The present paper assessed the efficacy of a food-based dietary fat exchange model, that replaced dietary SFAs with MUFAs and PUFAs in free-living UK men, with endpoint measures of nutrient intakes, overall dietary patterns and plasma PL-FAs.

## Methods

### Study design

The RISSCI-1 study was a single-blind sequential dietary intervention study (ClinicalTrials.Gov registration No. NCT03270527). The study was given a favourable ethical opinion for conduct by the University of Reading Research Health Ethics Committee (17/29) and the University of Surrey Ethics Committee (UEC/2017/41/FHMS) and was conducted in accordance with the Declaration of Helsinki guidelines. Written informed consent was collected from all participants before inclusion in the study.

### Participants

The RISSCI-1 study included healthy men aged 30–65 years, which were recruited from the Reading, Berkshire and Guildford, Surrey areas between 2017 and 2019. Eligible participants were required to meet the following inclusion criteria: body mass index (BMI) between 19 and 32 kg/m^2^; fasting serum total cholesterol < 7.5 mmol/L and triacylglycerol < 2.3 mmol/L; blood pressure < 140/90 mmHg; fasting glucose < 7.0 mmol/L; haemoglobin > 130 g/L; no history of myocardial infarction, stroke, diabetes, or any other endocrine disorder in the past 12 months; no history of kidney, liver, or gastrointestinal disorder, or history of cancer; not taking any medication for hyperlipidaemia, hypertension, inflammation, or prescribed antibiotics in the last three months; not smoking; drinking ≤ 14 units of alcohol per week; participating in vigorous exercise ≤ 3 times per week; not participating or planning to participate in a weight-loss diet; not taking any dietary supplements known to influence circulating lipids or gut microbiota (e.g. plant stanols, fish oil, phytochemicals, natural laxatives, probiotics and prebiotics); not being involved in another dietary intervention study and willing to regularly consume study intervention products (i.e. butter/spreads, oils, dairy foods, and snacks). Upon inclusion, participants were advised to maintain their usual physical activity levels, and to inform the researchers of any important changes to their health or medication use.

### Dietary intervention and food-exchange model

The replacement of dietary SFAs with MUFAs/PUFAs was based on a food-exchange model which was successfully implemented in previous intervention studies at the University of Reading [11–13]. The food-exchange model aimed to identify dietary sources of exchangeable fat that would not impact total energy or other macronutrient intakes. Estimated amounts of dietary exchangeable fat from oil, butter and fat spreads, dairy foods, and snacks were calculated using data from the National Diet and Nutrition Survey (NDNS) (y 1–4) in UK adult men aged 19–64 years [21], and the Dietary Intervention and Vascular function (DIVAS) randomised controlled trial (RCT) [12] (Table 1*)*. These estimates were then converted into servings of common commercially available cooking oils and fat spreads, dairy foods, and sweet and savoury snacks that participants were required to consume daily to achieve the nutrient targets in each dietary intervention period (Table 2).

**Table 1:** Identified sources of dietary exchangeable fat in the RISSCI-1 food-exchange model

	Total energy	Total fat		SFAs		MUFAs		PUFAs	
	MJ/d	g/d	%TE	g/d	%TE	g/d	%TE	g/d	%TE
Total baseline intake (including alcohol)^a^	8.80	77.7	32.8	28.4	11.9	28.5	12.0	13.4	5.7
Sources of exchangeable fat									
Added oils^b^	0.38	8.7	3.7	0.8	0.3	3.2	1.4	1.5	0.6
Added fats (butter and spreads)	0.29	7.8	3.3	2.8	1.2	2.9	1.2	1.4	0.6
Milk	0.44	4.3	1.8	2.7	1.2	1.1	0.5	0.1	< 0.1
Cheese	0.26	5.0	2.1	3.0	1.3	1.3	0.6	0.2	< 0.1
Sweet and savoury snacks^c^	0.86	9.9	4.2	3.8	1.6	3.4	1.5	1.6	0.7
Total exchangeable fat intake	2.15	35.8	15.3	13.1	5.6	12.0	5.1	4.8	2.1
Non-exchangeable fat intake	6.65	41.9	17.9	15.3	6.5	16.5	7.1	8.6	3.7

*%TE* % total energy, *MJ/d* megajoules/day, *SFAs* saturated fatty acids, *MUFAs* monounsaturated fatty acids, *PUFAs* polyunsaturated fatty acids, *RISSCI-1* Reading, Imperial, Surrey, Saturated fat Cholesterol Intervention-1

Adapted from Weech et al*.*[12]

^a^Calculation based on the National Diet and Nutrition Survey (year 1 to 4) in men aged 19–64 years [21]

^b^Calculation based on the Dietary Intervention and Vascular function (DIVAS) randomised controlled trial [12]

^c^Included biscuits, buns, cakes, pastries, fruit pies, savoury snacks, and chocolate

**Table 2:** Recommended daily servings of intervention food items for the achievement of the RISSCI-1 dietary fat exchange

Intervention food item	High-SFA diet		Low-SFA diet	
	Description	Recommended amount (g/d)	Description	Recommended amount (g/d)
Fat spreads	Salted butter^a^	14	Vegetable fat spread^a,b^	17
Cooking fats	Salted butter^a^	6	Sunflower oil^a^	11
Cheese or yogurt	Cheese with ≥ 25% fat, or full-fat yogurt	25 (cheese) or 100 (yogurt)	Cheese with < 25% fat, or virtually fat free yogurt	25 (cheese) or 100 (yogurt)
Milk	Full fat or semi-skimmed	200	< 1% fat	200
Snacks	Chocolates, biscuits, and crackers^a^	50	Crisps and nuts^a^	50

*SFA* saturated fatty acid, *MUFA* monounsaturated fatty acid, *PUFA* polyunsaturated fatty acid, *RISSCI-1* Reading, Imperial, Surrey, Saturated fat Cholesterol Intervention-1

^a^Food items provided by researchers. Items provided for the high-SFA diet included: Wyke Farms “Salted Butter”, Whitworths “Banana Chips”, McVitie’s “Gold Bar”, Mrs Crimble’s “Big Choc Macaroon”, McVitie’s “Trio Toffee Biscuit bar”, Sainsbury’s “Belgian Chocolate Chunk Shortbread”, Tunnock’s “Caramel Wafer”, Sainsbury’s “Cheddar Cheese Crispies”, Arden’s “Cream Cheese and Spring Onion Melts”, and Jacob’s “Savours Sweet Chilli Thins Crackers”. Items provided for the low-SFA diet included: Flora “Buttery Spread”, KTC “100% Sunflower Seed Oil”, Tesco “Crispy Seedy Nutty Bites”, Sainsbury’s “Unsalted Mixed Nuts and Raisins”, Tesco “Sweet Chilli Coated Peanuts”, Sesame Snaps^®^, Tesco “Bombay Mix”, Nik Naks “Nice & Spicy Corn Snacks”, Tesco “Ready Salted Crisps”, Walkers “Max Paprika Crisps”, and Pringles “Original Crisps”

^b^79% vegetable fat spread with 5% sunflower oil and 24% rapeseed oil

To achieve the exchange of dietary fat, the RISSCI-1 sequential dietary intervention consisted of two, 4-week, isoenergetic, moderate-fat diets (34% TE from fat). The first intervention period was a high-SFA diet (target%TE SFA:MUFA:PUFA = 18:12:4), and the second intervention period was a low-SFA, high-MUFA/PUFA diet (target%TE SFA:MUFA:PUFA = 10:14:10). Both 4-week diets were otherwise broadly matched for other macronutrients, and aimed to comply with the COMA 1991 recommendations which stated n-6 PUFAs should not exceed 10%TE [7]. To reproduce a transition from a high intake of SFA to the lower intake representative of the UK public health guideline for SFA intake of no more than 10%TE with recommendations to replace with unsaturated fats, all participants received the high-SFA diet for the first 4-week period, followed by the low-SFA, high-MUFA/PUFA diet for the second 4-week period without a washout period.

### Implementation of intervention diets

Participants were invited to attend three study visits: at baseline upon inclusion (week 0), after completing the high-SFA diet (week 4), and low-SFA diet (week 8). At the first two study visits, participants were provided with a detailed information booklet containing instructions on how to comply with the high-SFA or low-SFA dietary guidelines, along with tailored recommendations to suit their lifestyle (e.g. meals out of the home, cooking for the family meal ideas and recipes). To improve compliance, participants also received free-of-charge study food items to incorporate into their baseline diets. Supplied food items included fat spreads, cooking oils, and an assortment of sweet and savoury snacks in sufficient quantity for each 4-week dietary intervention period. Due to their shorter shelf-life, dairy foods such as milk and cheese were not supplied, and participants were instructed to purchase these foods. All the intervention foods were commercially available from major UK supermarkets.

To ensure compliance to dietary guidelines, each dietary intervention period was scheduled outside of major holiday periods (e.g. Christmas and Easter), and participants were required to avoid any extended periods away from their home. Participants were also asked to return any leftover study items from the high-SFA diet before starting the low-SFA dietary intervention period. To help incorporate the study foods into their usual diet, and to assess compliance, participants were provided with daily tick sheets to be completed throughout each intervention period. Participants were free to consume the provided food items either as part of their main meals or at any other time of day. Participants were also permitted to consume more than the minimum required daily servings of any study food items, if their habitual intake exceeded the recommended amount for the intervention and if they were maintaining a stable body weight (± 1 kg from week 0). The importance of the latter was emphasised to the participants at follow-up visits at the mid-point of each dietary intervention (weeks 2 and 6). During these short visits, daily tick sheets were reviewed, and participants were supplied with any additional study food items required to complete the remainder of the intervention period. If body weight varied by greater than 1 kg from baseline or the previous study visit, participants were advised to reduce or increase their consumption of the provided snacks or other food items as appropriate.

### Collection of dietary data

Participants were instructed to complete a 4-day weighed diet diary, a week before each study visit, to assess their baseline, habitual dietary intake (week -1), and during each dietary period to assess compliance to the interventions (weeks 3–4 and 7–8). Each diet diary included 3 weekdays and 1 weekend day during which participants were provided with digital scales to record the amount and description of all food items and beverages consumed. To improve the accuracy of the diet diaries, participants received additional diary templates to record all individual ingredients used in homemade recipes, along with published food portion tables to record foods consumed outside of the home [22]. Researchers assessed the completion and accuracy of the diet diary during each study visit, and requested any additional information necessary to improve data entry precision.

Paper diet diaries were analysed using Nutritics Research Edition v5.64 (Dublin, 2019) to assess foods consumed and nutrient intakes. Every item consumed was matched to its closest equivalent in the McCance and Widdowson’s Composition of Foods Integrated Dataset (CoFID) [23], which was used to calculate daily dietary consumptions of total energy, and selected macro- and micro-nutrients: protein, carbohydrate, free sugars, Association of Analytical Chemists (AOAC) fibre, alcohol, total fat, SFAs, MUFAs, PUFAs, n-3 PUFAs, n-6 PUFAs, *trans* fatty acids (TFAs), cholesterol, and sodium. In addition, researchers used the NDNS Rolling Programme nutrient databank to impute missing values of n-3/n-6 PUFAs in food items contributing to at least 1 g of PUFAs in each diet diary [21]. Food items consumed (in g/d) were classified into 40 food categories (supplementary Table 1), which were used to assess dietary patterns.

### Assessment of underestimation of energy consumption

Underestimation of dietary TE at baseline and during each dietary intervention periods was checked using the method proposed by Black [24]. Researchers estimated the basal metabolic rate of each participant using the Henry equations for men, based on age and body weight [25]. On the basis of a sedentary lifestyle (physical activity level score of 1.2 [12]), the lower 95% confidence limit of the Goldberg cut-off to identify under-reporters of dietary TE was estimated to lie between 1.13 and 1.16.

### Phospholipid fatty acid analyses

Blood was collected into EDTA vacutainers after an overnight fast (12 h) at baseline (week 0) and at the end of each dietary intervention period (weeks 4 and 8). After collection, vacutainers were chilled on ice for 20 min before centrifugation at 1750*g* (3000 rpm) for 15 min at 4 °C for the collection of plasma, which was stored at − 80 °C before subsequent analysis.

The extraction of fatty acids methyl esters (FAME) from plasma PL was performed using a 3-step protocol (i.e. lipid extraction, solid phase extraction and transmethylation) based on methods from Metges et al*.* [26], Kaluzny et al. [27], and Baylin et al*.* [28]. Briefly, plasma lipids were extracted using a tert-butyl methyl ether (MTBE)/methanol solution and PL were eluted in methanol using solid phase extraction on aminopropyl-silica columns (Chromabond, MachereyNagel GmbH & Co. KG, Düren, Germany). Dried PL were then suspended in 200 μL of toluene and 15 µL of trimethyl sulfonium hydroxide solution (TMSH, 0.2 mol/L in methanol, Macherey–Nagel, 701 520.101) to obtain fatty acid methyl esters. FAMEs were separated using a gas chromatograph (GC) (Agilent 7890A, Agilent Technologies, Waldbronn, Germany) and flame ionization detector (FID) equipped with a 100 m capillary column (HP-88, 100 m × 0.25 mm I.D., 0.2 µm film thickness, Agilent). Finally, FAMEs were identified against a standard mixture of 27 FAMEs (Supelco™) containing FAMEs of chain-length between C4 and C24. In subsequent analyses, fatty acid concentrations were calculated as weight percentage of total fatty acids detected (wt%). Inter-assay coefficients of variation (*n* = 10) were all below 6.4% (range 0.5–6.4%).

### Measurement of anthropometrics and physical activity levels

The evening before each study visit (weeks 0, 4, and 8), participants were asked to consume a supplied, low-fat meal (< 1.5 MJ and < 7 g total fat content) with low-nitrate water (Buxton Mineral Water, Nestlé Waters, Buxton, UK) and to fast overnight for at least 12 h consuming only the low-nitrate water provided. On the morning of the study visit, researchers recorded height (to the nearest 0.1 cm), body weight (to the nearest 0.1 kg), and calculated the BMI of each participant using a wall-mounted stadiometer and a Tanita BC-418 (Reading) or Tanita BC-420MA (Surrey) digital scale (Tanita Europe). An allowance of 1 kg was included for light clothing when assessing body weight, and the digital scale was operated under the “standard body type” setting. Physical activity habits were assessed through the participants’ completion of the long version of the International Physical Activity Questionnaire (IPAQ), and physical activity levels were classified into three categories (i.e. “Low”, “Moderate”, and “High”) using the IPAQ guidelines for categorisation [29].

### Power calculations and statistical analyses

A required sample size of 92 participants was estimated for the detection of a 0.16 mmol/L (SD 0.54) difference in fasting LDL-C concentrations (primary outcome in the main RISSCI-1 study) between the high- and low-SFA diets, as observed in the DIVAS parallel RCT [30], with an 80% statistical power and a 5% significance level. After accounting for a 15% dropout rate, this increased to a total of 106 participants. A sample size of 106 participants was also adequate for the investigation of PL-FA responses to the interventions. In this study, the successful replacement of dietary SFAs with MUFAs/PUFAs was expected to decrease the abundance of total SFAs in plasma PL-FAs by an estimated 0.46% of area of total PL-FAs (SD 0.8) [12], leading to a required sample size of 30 participants (i.e. *n* = 26 participants for a detection with an 80% statistical power and a 5% significance levels, and *n* = 4 participants to allow for a 15% dropout).

Since the RISSCI-1 dietary intervention was isoenergetic, the stability of BMI throughout the intervention was assessed using a linear mixed model which included age (continuous, y), study visit (week 0, week 4, or week 8), and study centre (University of Reading, University of Surrey) as fixed effects, and participants as a random effect. Daily average nutrient intakes from 4-day diet diaries and plasma PL-FA concentrations were compared between the high-SFA diet (week 4) and the low-SFA diet (week 8) using paired *t* tests. All variables were checked for normality and log-transformed if necessary. In the case of alcohol consumption, *t* tests were performed on alcohol consumers only and non-consumers were excluded from statistical analyses.

Furthermore, food categories and plasma PL-FA concentrations during the high-SFA and low-SFA diet were analysed using orthogonal partial least square discriminant analyses (OPLS-DA) to identify dietary patterns and circulating FA profiles in response to the RISSCI-1 dietary intervention [31, 32]. All variables were mean-centered and divided by their standard deviation (SD). Statistical significance of the OPLS-DA models was tested using internal cross-validation permutation tests (*n* = 1000 permutations), and goodness of fit and predictive accuracy were assessed using the R^2^Y and Q^2^ values, respectively. For the interpretation of the models, variable loadings scaled as correlations towards the predictive model (*p*(corr)) were used to identify the variables that contributed the most to the discrimination of dietary patterns or plasma PL-FA profiles between the high-SFA and the low-SFA diets.

In exploratory analyses, a constraint-based feature selection algorithm was used to identify plasma PL-FAs associated with dairy fat consumption [33]. This method is based on a forward–backward feature selection approach and aims to reduce the dimension of a given dataset by providing multiple statistically equivalent subsets of features with maximised predictive accuracy. In prospective analyses, plasma PL-FA concentrations were calculated as changes between the high-SFA diet (week 4), which was enriched in full-fat dairy foods, and baseline (week 0). In addition, cross-sectional analyses aimed to identify predictors of baseline dairy fat consumption among baseline concentrations of plasma PL-FAs. In both approaches, selected predictors among plasma PL-FAs were fitted in multiple linear regression models with adjustments for age (y), BMI (kg/m^2^), baseline dairy fat consumption (g/d, in prospective models only), and energy intakes at baseline (kcal/d). Predictive R^2^ coefficients were used to assess the predictive accuracy of multiple linear regression models.

All statistical analyses were conducted in R (version 4.0.4), except from OPLS-DA models which were fitted in MetaboAnalyst version 5.0 [34].

## Results

**Fig. 1:**
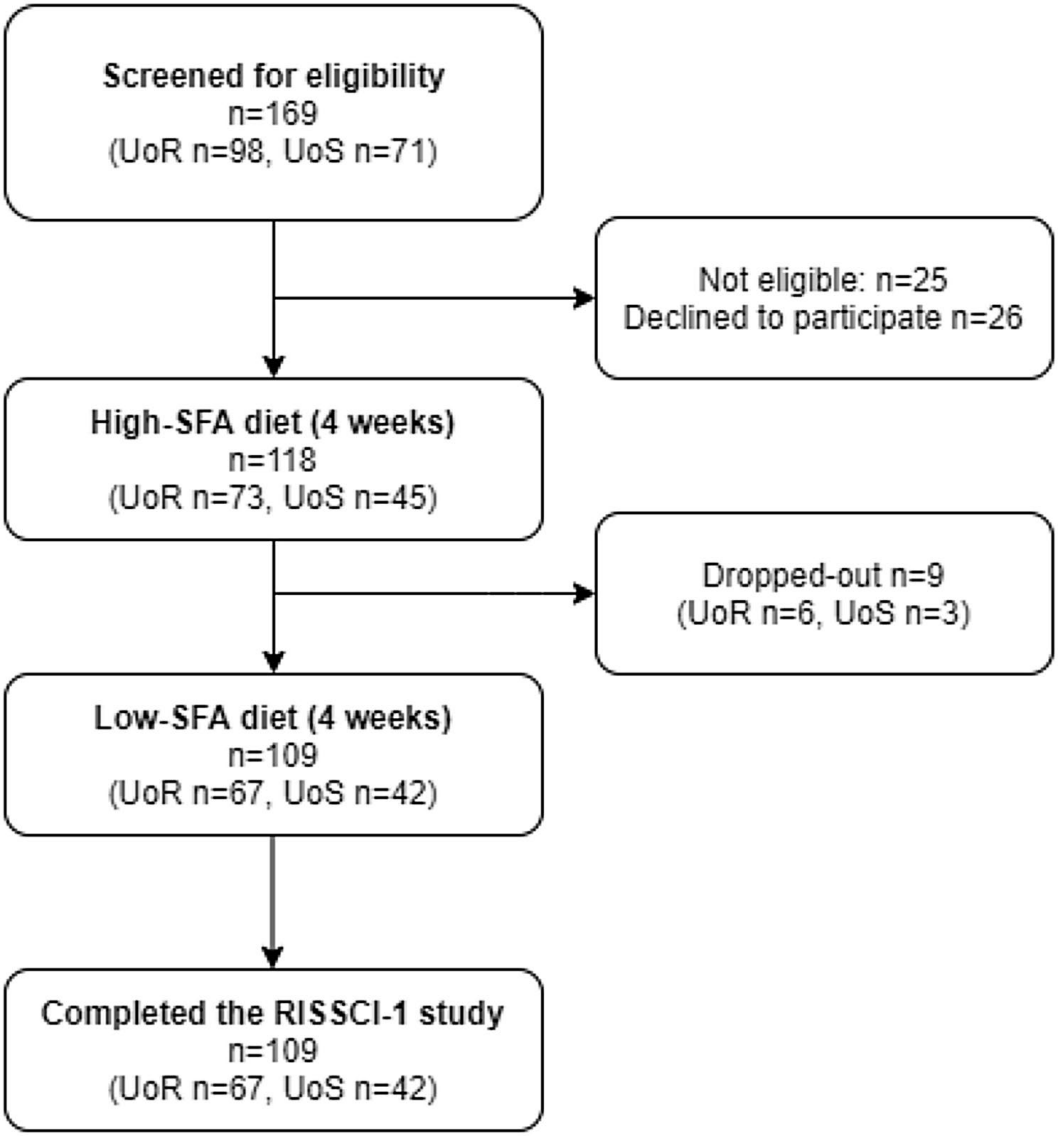
Flow-chart of participants from the RISSCI-1 study. *UoR* University of Reading, *UoS* University of Surrey

The flowchart of participants included in the RISSCI-1 study is presented in Fig. 1. A total of 118 participants were enrolled to follow the first dietary intervention period (i.e. high-SFA diet), including 9 participants who withdrew from the study at the end of the first diet (*n* = 6 due to time or work commitments, *n* = 2 due to loss of interest in the study, *n* = 1 due to newly prescribed medication). The remaining 109 participants completed both the first (high-SFA) and second dietary intervention period (low-SFA diet), giving an overall dropout rate of 7.6%.

Baseline characteristics of participants are presented in Table 3. Participants mean age was 48 y (SD 11), with a BMI of 25.1 kg/m^2^ (SD 3.3). Participants were of Asian or UK Asian (7.3%), Black or UK Black (2.8%), Chinese (1.8%), Mixed Ethnic (1.8%), or White (86.2%) self-reported ethnic backgrounds. Finally, most participants had moderate or high, self-reported physical activity levels (31.2% and 47.7%, respectively).

**Table 3:** Baseline characteristics of adult men from the RISSCI-1 study (*n* = 109)

	Mean	SD
Age, y	48.4	10.8
Self-reported ethnicity, *n* (%)		
Asian or UK Asian	8 (7.3)	
Black or UK Black	3 (2.8)	
Chinese	2 (1.8)	
Mixed Ethnic Background (not specified)	2 (1.8)	
White	94 (86.2)	
BMI, kg/m^2^	25.1	3.3
Physical activity level, *n* (%)^a^		
Low	6 (5.5)	
Moderate	34 (31.2)	
High	52 (47.7)	
Missing	17 (15.6)	
Total energy		
kcal/d	2320	635
MJ/d	9.7	2.7
Total fat, %TE	36.2	7.8
SFAs, %TE	12.7	3.8
MUFAs, %TE	13.3	3.5
n-3 PUFAs, %TE	0.8	0.4
n-6 PUFAs, %TE	4.6	1.8
Total PUFAs, %TE	5.8	2.1
TFAs, %TE	0.5	0.3
Cholesterol, mg/d	235	116
Protein, %TE	16.3	3.3
Carbohydrates, %TE	44.3	9.4
Free sugars, %TE	7.6	4.8
Dietary fibre (AOAC), g/d	25.8	9.5
Alcohol, %TE ^b^	4.0	(1.4–7.7)
Sodium, g/d	2.6	1.0

*AOAC* Association of Analytical Chemists, *BMI* body mass index, *d* day, *MUFAs* monounsaturated fatty acids, *PUFAs* polyunsaturated fatty acids, *RISSCI-1* Reading, Imperial, Surrey, Saturated fat Cholesterol Intervention-1, *SD* standard deviation, *SFAs* saturated fatty acids, *TFAs* trans fatty acids ,%*TE* % total energy

^a^Categories derived from the International Physical Activity Questionnaire (IPAQ) [29]

^**b**^Values presented as *median (interquartile range)* and based on *n* = 45 participants who consumed alcohol (*n* = 55 non-consumers)

### Dietary consumption

Nutrient intakes during each dietary intervention period are shown in Table 4. Out of the 109 participants who completed the RISSCI-1 study, nine were excluded from the dietary analyses due to insufficient or incomplete dietary data. There were no significant differences between the dietary energy, macronutrients (total fat, carbohydrates, and proteins), AOAC dietary fibre or alcohol consumption during the high-SFA and low-SFA diets. Data on average daily nutrient consumption indicated a successful exchange of dietary SFAs for MUFAs and PUFAs during the second dietary intervention period, with dietary SFA consumption decreasing from 19.1%TE (SD 3.5) during the high-SFA diet to 8.9%TE (SD 2.1) during the low-SFA diet (*p* < 10^–3^). The observed decrease in SFA intake was compensated for by a rise in MUFA and PUFA consumptions from 11.1%TE (SD 2.8) and 3.7%TE (SD 1.3), respectively during the high-SFA diet to 13.4%TE (SD 2.9), and 11.1%TE (SD 3.6) during the low-SFA diet (both *p* < 10^–3^). In addition, participants consumed less TFAs (*p* < 10^–3^), dietary cholesterol (*p* < 10^–3^), and sodium (*p* = 0.04) during the low-SFA diet compared to the high-SFA diet.

**Table 4:** Recorded and target daily nutrient intakes following each dietary intervention period (high-SFA and low-SFA diets) in adult men from the RISSCI-1 study (*n* = 100)

	High-SFA diet			Low-SFA diet			
	Target	Mean	SD	Target	Mean	SD	*p*^a^
Total energy							
kcal/d		2354	546		2282	558	0.13
MJ/d		9.9	2.3		9.6	2.3	0.14
Total fat, %TE	34.0	38.4	6.5	34.0	38.2	6.6	0.79
SFAs, %TE	18.0	19.1	3.5	10.0	8.9	2.1	< 10^–3^
MUFAs, %TE	12.0	11.1	2.8	14.0	13.4	2.9	< 10^–3^
*n*−3 PUFAs, %TE		0.6	0.4		1.2	0.5	< 10^–3^
*n*−6 PUFAs, %TE		2.5	1.0		9.5	3.5	< 10^–3^
Total PUFAs, %TE	4.0	3.7	1.3	10.0	11.1	3.6	< 10^–3^
TFAs, %TE		0.8	0.3		0.2	0.2	< 10^–3^
Cholesterol, mg/d		273	112		201	166	< 10^–3^
Protein, %TE		16.0	3.0		16.3	3.1	0.28
Carbohydrates, %TE		42.6	7.9		42.9	8.0	0.61
Free sugars, %TE		5.0	3.9		4.7	3.2	0.35
Dietary fibre (AOAC), g/d		24.4	10.3		25.9	11.9	0.06
Alcohol, %TE^b^		4.5	(2.2–6.2)		3.6	(2.0–5.6)	0.83^c^
Sodium, g/d		2.67	0.88		2.45	0.91	0.04

*AOAC* Association of Analytical Chemists, *d* day, *MUFAs* monounsaturated fatty acids, *PUFAs* polyunsaturated fatty acids, *RISSCI-1* Reading, Imperial, Surrey, Saturated fat Cholesterol Intervention-1, *SD* standard deviation, *SFAs* saturated fatty acids, *TFAs* trans fatty acids, *%TE* % total energy

Means and SD based on *n* = 100 participants, unless specified otherwise

^**a**^From paired *t* tests

^**b**^Values presented as *median* (interquartile range) and based on *n* = 45 participants who consumed alcohol (*n* = 55 non-consumers)

^**c**^From paired *t* test on log-transformed values between the high-SFA and low-SFA diets

### Energy balance

There was no statistically significant impact of the dietary interventions on participants’ BMI (*p* = 0.7 for the high-SFA diet, and 0.1 for the low-SFA diet, compared to baseline). Estimated marginal means for BMI at baseline, following the high-SFA diet, and following the low-SFA diet were 25.1 kg/m^2^ (95% CI 24.4–25.7), 25.1 kg/m^2^ (95% CI 24.4–25.7), and 25.0 kg/m^2^ (95%CI 24.4–25.7), respectively. The proportions of under-reporters of energy intake at baseline, following the high-SFA diet and following the low-SFA diet were estimated at 28%, 17%, and 27%, respectively, based on the assumption that participants remained in energy balance throughout the study.

### Analysis of plasma PL-FAs

Relative concentrations of plasma PL-FAs after each 4-week dietary intervention period are shown in Table 5. All plasma PL-FA concentrations were significantly different between the high-SFA and low-SFA diets apart from those of elaidic acid (18:1 n−9 *trans*), γ-linolenic acid (18:3 n−6), and α-linolenic acid (18:3 *n*−3). Overall, plasma PL samples after the low-SFA diet had lower abundances of 16 individual and classes of plasma PL-FAs which included palmitic acid (16:0, difference between high-SFA and low-SFA diet (Δ) = − 1.23 wt%, *p* < 10^–4^), total SFAs (Δ = − 0.84 wt%, *p* < 10^–4^), *n*−3 PUFAs (Δ = − 0.52% total FA, *p* < 10^–4^), dihomo-γ-linolenic acid (20:3 n−6, Δ = − 0.41 wt%, *p* < 10^–4^), and total MUFAs (Δ = − 0.31 wt%, *p* < 10^–2^), but higher abundances of 10 individual and classes of plasma PL-FAs, which included linoleic acid (18:2 *n*−6, Δ = 1.87 wt%, *p* < 10^–4^), *n*−6 PUFAs (Δ = 1.69 wt%, *p* < 10^–4^), total PUFAs (Δ = 1.15 wt%, *p* < 10^–4^), stearic acid (18:0, Δ = 0.53 wt%, *p* < 10^–4^), and arachidonic acid (20:4 *n*−6, Δ = 0.31 wt%, *p* < 10^–2^).

**Table 5:** Fasting abundances of plasma phospholipid fatty acids following the low-SFA and high-SFA diets in adult men from the RISSCI-1 study (*n* = 108)

Fatty acid abundances (wt%)	High-SFA Diet		Low-SFA Diet		Δ^a^		
	Mean	SD	Mean	SD	Mean	SD	*p*^b^
Total SFAs	46.0	0.9	45.1	1.1	− 0.84	0.90	< 10^–4^
14:0	0.55	0.12	0.46	0.11	− 0.09	0.12	< 10^–4^
15:0	0.28	0.05	0.21	0.04	− 0.06	0.04	< 10^–4^
16:0	30.3	1.2	29.0	1.3	− 1.23	1.20	< 10^–4^
17:0	0.44	0.06	0.42	0.06	− 0.02	0.04	< 10^–4^
18:0	14.3	1.0	14.9	1.0	0.53	0.74	< 10^–4^
20:0	0.09	0.01	0.11	0.02	0.02	0.02	< 10^–4^
22:0	0.03	0.01	0.03	0.01	0.01	0.01	< 10^–4^
Total MUFAs	12.6	1.3	12.3	1.3	− 0.31	1.11	< 10^–2^
16:1 *n*−7 *cis*	0.52	0.21	0.42	0.18	− 0.09	0.13	< 10^–4^
18:1 *n*−9 *cis*	10.2	1.2	9.9	1.2	− 0.26	1.02	< 10^–2^
18:1 *n*−7 *cis*	1.43	0.20	1.49	0.22	0.06	0.17	< 10^–4^
20:1 *n*−9	0.18	0.04	0.23	0.05	0.05	0.04	< 10^–4^
16:1 *n*−7 *trans*	0.01	0.00	0.01	0.00	− 0.003	0.004	< 10^–4^
18:1 *n*−9 *trans*	0.15	0.04	0.15	0.04	0.004	0.040	0.37
18:1 *n*−7 *trans*	0.18	0.06	0.11	0.04	− 0.07	0.06	< 10^–4^
Total PUFAs	41.4	1.6	42.5	1.6	1.15	1.34	< 10^–4^
20:3 *n*−9	0.15	0.04	0.13	0.04	− 0.02	0.05	< 10^–3^
Total PUFAs *n*−6	35.5	2.1	37.1	2.0	1.69	1.73	< 10^–4^
18:2 *n*−6 *cis*	21.4	2.5	23.2	2.4	1.87	1.75	< 10^–4^
18:3 *n*−6	0.09	0.05	0.09	0.05	− 0.005	0.041	0.26
20:2 *n*−6	0.33	0.05	0.34	0.06	0.01	0.05	0.01
20:3 *n*−6	3.38	0.83	2.97	0.74	− 0.41	0.52	< 10^–4^
20:4 *n*−6	9.70	1.71	9.99	1.86	0.30	0.97	< 10^–2^
22:4 *n*−6	0.35	0.08	0.32	0.09	− 0.03	0.04	< 10^–4^
22:5 *n*−6	0.20	0.06	0.16	0.06	− 0.04	0.03	< 10^–4^
18:2 *n*−6 *trans*	0.06	0.01	0.06	0.01	− 0.002	0.007	< 10^–2^
Total PUFAs *n*−3	5.76	1.49	5.23	1.19	− 0.52	0.92	< 10^–4^
18:3 *n*−3	0.22	0.07	0.22	0.08	0.00	0.07	0.53
20:5 *n*−3	1.25	0.69	0.99	0.53	− 0.26	0.48	< 10^–4^
22:5 *n*−3	1.08	0.20	0.95	0.20	− 0.12	0.14	< 10^–4^
22:6 *n*−3	3.21	0.90	3.07	0.79	− 0.14	0.50	< 10^–2^

*MUFAs* monounsaturated fatty acids, *PUFAs* polyunsaturated fatty acids, *RISSCI-1* Reading, Imperial, Surrey, Saturated fat Cholesterol Intervention-1, *SD* standard deviation, *SFAs* saturated fatty acids, *wt%* weight percentage of total fatty acids

^a^Δ = low-SFA – high-SFA values

^b^From paired *t* tests

**Fig. 2:**
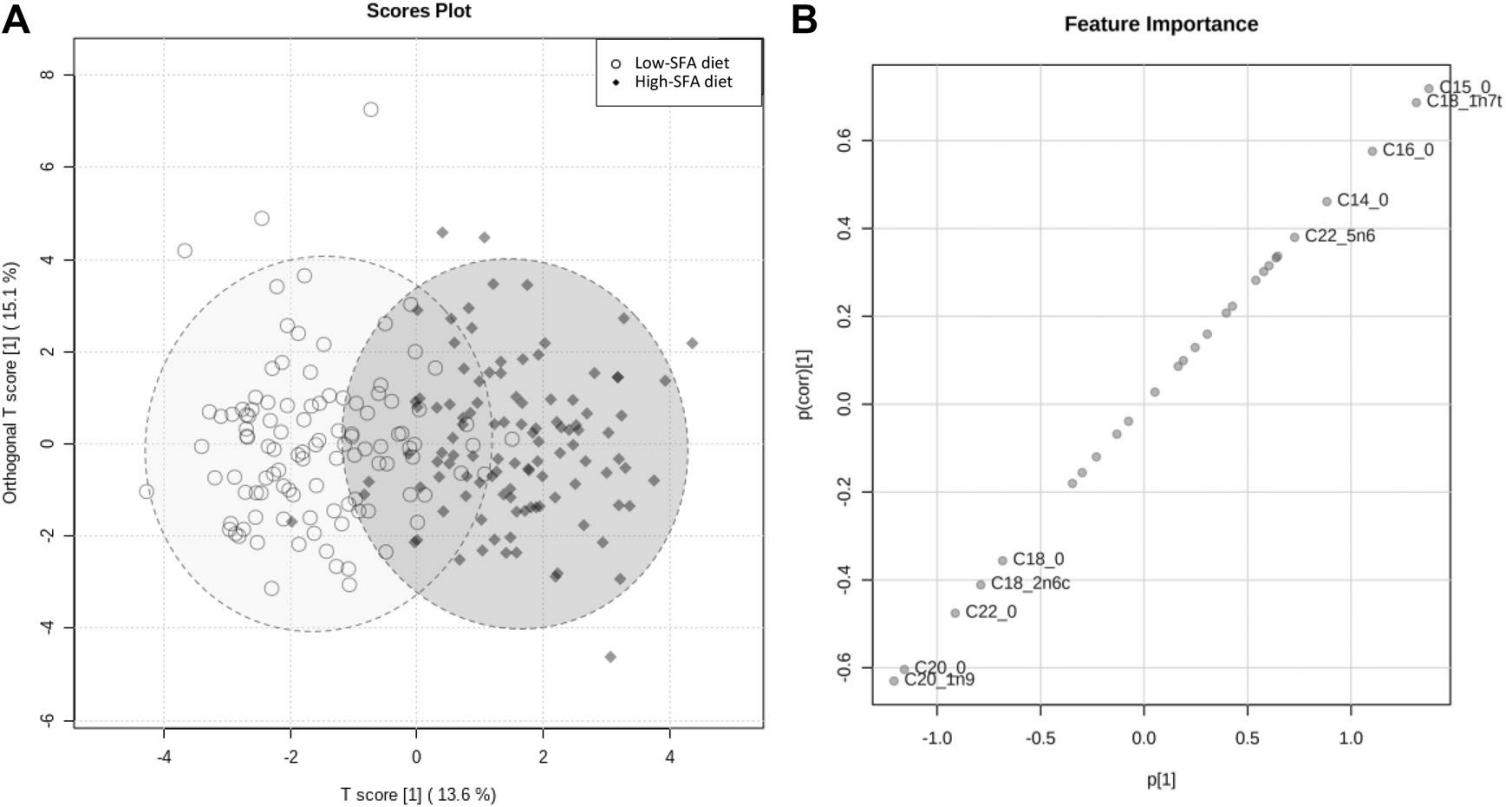
Orthogonal Partial Least Square Discriminant Analysis (OPLS-DA) based on plasma phospholipid fatty acids (PL-FAs) in adult men from the RISSCI-1 study between the high-SFA and the low-SFA diets (*n* = 108). **A** Scores plot showing a moderate discrimination between two PL-FA profiles during the high-SFA and low-SFA diets. **B** Feature loadings scaled as correlation coefficients (*p*(corr)[1]) towards the OPLS-DA predictive component (*p*[1]), showing the individual PL-FAs contributing to each discriminated FA profile

In OPLS-DA of the plasma PL-FA abundances during the high-SFA and low-SFA diets, the first component of the model, which explained 13.6% of the total variation, was retained for interpretation (Fig. 2A). The OPLS-DA, which aimed to discriminate plasma PL-FA profiles specific to each dietary intervention period, revealed moderate fitness (R^2^Y = 0.66, empirical permutation *p* < 0.01 (0/1000)) and predictive accuracy (Q^2^ = 0.57, empirical permutation *p* < 0.01 (0/1000)). As shown in Fig. 2B, discriminating plasma PL-FAs during the high-SFA diet included pentadecanoic acid (15:0, p(corr) = 0.72), *trans* vaccenic acid (18:1 *n*−7 *trans*, *p*(corr) = 0.69), palmitic acid (16:0, *p*(corr) = 0.58), myristic acid (14:0, *p*(corr) = 0.46), and *n*−6 docosapentaenoic acid (22:5 *n*−6, p(corr) = 0.38). In contrast, the low-SFA plasma PL-FA profile showed higher abundances of eicosenoic acid (20:1 *n*−9, *p*(corr) = − 0.63), arachidic acid (20:0, *p*(corr) = − 0.60), behenic acid (22:0, *p*(corr) = − 0.48), linoleic acid (18:2 *n*−6, *p*(corr) = − 0.41), and stearic acid (18:0, *p*(corr) = − 0.36).

### Analysis of dietary patterns

**Fig. 3:**
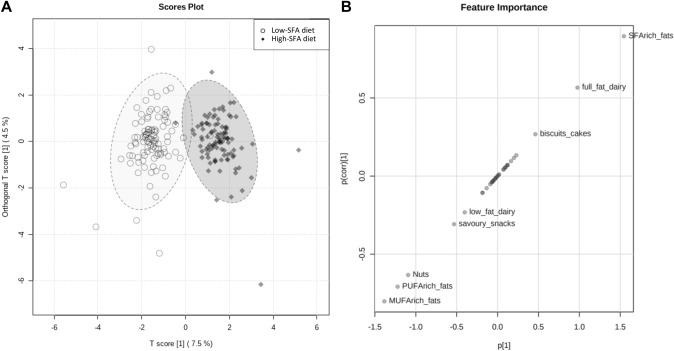
Orthogonal Partial Least Square Discriminant Analysis (OPLS-DA) based on dietary intakes in adult men from the RISSCI-1 study between the high-SFA and the low-SFA diets (*n* = 100). **A** Scores plot showing the discrimination between two dietary patterns during the high-SFA and low-SFA diets. **B** Feature loadings scaled as correlation coefficients (*p*(corr)[1]) towards the OPLS-DA predictive component (*p*[1]), showing the food groups contributing to each discriminated dietary pattern

For the recorded consumption of 40 food categories during the high-SFA and low-SFA diets, the first component of the model (OPLS-DA) was retained for the discrimination of dietary patterns during the two diets, and explained 7.5% of the overall variation (Fig. 3A). The retained model showed adequate fitness (R^2^Y = 0.82, empirical permutation *p* < 0.01 (0/1000)) and predictive accuracy (Q^2^ = 0.68, empirical permutation *p* < 0.01 (0/1000)). As shown in Fig. 3B, the high-SFA dietary pattern was characterised by higher intakes of SFA-rich fat (correlation scaled loading *p*(corr) = 0.89), full-fat dairy foods (*p*(corr) = 0.57), and biscuits and cakes (*p*(corr) = 0.27). In contrast, the low-SFA dietary pattern was characterised by higher consumptions of MUFA-rich fat (*p*(corr) = − 0.80), PUFA-rich fat (*p*(corr) = − 0.71), nuts (*p*(corr) = − 0.63), savoury snacks (*p*(corr) = − 0.31), and low-fat dairy (*p*(corr) = − 0.23). Other food categories, such as cereals and grains, meats, fish, or fruits and vegetables, did not contribute significantly to the dietary pattern discrimination between the low-SFA and high-SFA diets.

### Associations between dairy consumption and plasma PL-FAs

In accordance with the dietary fat exchange model developed for the RISSCI-1 study (Table 1), dietary intakes from the 4-day weighed diet diaries showed that total dairy foods were important contributors of total fat (39.6%, SD 11.5) and SFA consumption (50.1%, SD 12.6) during the high-SFA diet compared to baseline (16.6% SD 11.4 for total fat, and 28.5% SD 17.5 for SFA) (supplementary Table 2).

Prospective constraint-based feature selection analyses identified two independent predictors of changes in dairy fat consumption among plasma PL-FAs after the high-SFA diet compared to baseline: pentadecanoic acid (15:0) and *trans* vaccenic acid (18:1 *n*−7 *trans*). In prospective multiple linear regression models between the end of the high-SFA diet and baseline (*n* = 104 participants), each additional 1% (%wt total FA) of pentadecanoic acid abundance in PL-FAs was associated with a 158 g/d increase in the reported intake of dairy fat (95% CI 81–235, *p* < 10^–3^). In a separate linear regression model, each additional unit of circulating *trans* vaccenic acid was associated with an increase of 84 g/d of reported dairy fat intake (95% CI 26–142, *p* = 0.005). In addition, the linear regression model based on pentadecanoic acid abundance had a slightly better predictive accuracy (predictive *R*^2^ = 0.27) than the model based on *trans* vaccenic acid (predictive *R*^2^ = 0.21). In cross-sectional analyses of baseline data (*n* = 106), pentadecanoic acid and *trans* vaccenic acid were also identified as two independent predictors of dairy fat consumption. However, linear regression models for both pentadecanoic acid (β = 92 g/d of reported dairy fat, 95% CI 42–142, *p* < 10^–3^) and *trans* vaccenic acid (*β* = 100 g/d of reported dairy fat, 95% CI 50–150, *p* < 10^–3^) showed weaker prediction accuracy, compared to prospective models (predictive *R*^2^ = 0.10 for pentadecanoic acid, and 0.12 for *trans* vaccenic acid).

## Discussion

The analyses of 4-day weighed diet diaries and plasma PL-FA profiles confirmed that the participants reached the nutritional targets set in our model, by reducing their consumption of dietary SFAs by 10.2%TE from the high-SFA diet to the low-SFA diet. This decrease in SFAs was mostly compensated by an increase in dietary MUFAs and PUFAs by 2.3%TE and 7.4%TE, respectively, while maintaining other macronutrient intakes. The exchange of dietary SFAs for UFAs was achieved without affecting total energy intake or BMI, which confirmed that participants remained in energy balance throughout the study. In addition, discriminant analyses of dietary patterns constituted a novel method of confirming compliance to the RISSCI-1 dietary guidelines, by showing that participants integrated the recommended and supplied study foods into their diets to exchange dietary SFAs for UFAs, without modifying their overall dietary patterns (e.g. via changes in intakes of meat, fish, cereals and grains, fruits, and vegetables).

The analysis of plasma PL-FAs during the two dietary intervention periods provides further evidence in support of the successful implementation of the RISSCI-1 dietary fat exchange, by revealing a 0.84 wt% decrease in total SFAs, 0.31 wt% decrease in total MUFAs, and 1.15 wt% increase in total PUFAs during the low-SFA compared to the high-SFA diet. The rise in plasma PL PUFAs during the low-SFA diet was driven by n-6 PUFAs (1.70 wt% increase), whereas circulating n-3 PUFAs decreased by 0.53 wt%. These results reflect the type of dietary fat consumed during the two diets, albeit on a much smaller scale, and with the caveat that even-chain SFAs and UFAs are subject to endogenous synthesis and oxidation in humans, limiting their reliability and utility as biomarkers of fat consumption [35]. In this respect, it is noteworthy that while total circulating palmitic acid has been reported to be associated with dietary intakes of carbohydrates and alcohol [36, 37], intakes of these macronutrients in the current study were not significantly different between the diets.

Furthermore, dietary analyses revealed small but significantly higher intakes of dietary TFAs and cholesterol during the high- compared to the low-SFA diet (decreases in 0.6%TE and 72 mg during the low-SFA diet, respectively). Since the abundance of elaidic acid (a *trans* FA mostly found in industrially processed food) in plasma PL did not differ between the high- and low-SFA diets, these differences may be explained by the guidelines to consume full-fat dairy foods and butter during the high-SFA diet, which contain naturally occurring ruminant *trans* FAs and cholesterol [38, 39]. However, participants remained well below the dietary reference value for TFAs of 2%TE [7], and small variations in dietary cholesterol (i.e. equivalent to less than that from a single egg yolk [23]) are unlikely to impact on plasma LDL-C. Moreover, current epidemiological evidence suggests that TFAs from dairy may not be associated with deleterious cardiometabolic outcomes as opposed to industrial TFAs [40, 41]. Similarly, higher sodium intakes were observed during the high-SFA diet compared to the low-SFA diet. This may reflect the dietary guidelines for this diet, which recommended daily servings of salted butter and cheese with higher salt content (e.g. Cheddar and Red Leicester) than those recommended during the low-SFA diet (e.g. cottage cheese and spreadable cream cheese). On average, study participants exceeded UK dietary recommendations for sodium of 2.4 g/d (6 g/d salt) at baseline and throughout the RISSCI-1 dietary intervention, but remained below the national average for men aged 19–64y which was estimated at 3.7 g/d (SD 1.7) in 2020 [42].

The dietary fat exchange model developed for this study used dairy as a key food group for the exchange of dietary SFA. Indeed, dairy foods represent an important entry point for SFA in the food chain as on average, they contribute 21% of dietary SFA intake in UK adults [14]. Nonetheless, and despite their SFA content, epidemiological evidence suggest an inverse or neutral association between dairy food consumption and cardiometabolic disease risk [43, 44]. This may stem from beneficial components and food matrix effects specific to some types of dairy food, such as bioactive peptides, fermentation process, or calcium-dependent fat sequestration [45]. These effects have not been demonstrated with butter, which may explain the detrimental associations observed between its consumption and cardiometabolic health outcomes [46, 47]. Apart from butter, other sources of dietary SFA, such as red and processed meat products, may have detrimental effects on cardiovascular health [48, 49] and were considered for the development of the previously implemented dietary fat exchange models [11]. However, a meat-based exchange of SFA was not achievable without compromising isoenergetic and equivalent macronutrient target intakes. In this context, the use of low-fat dairy products to reduce dietary SFA intakes in this study presents several advantages, as it helped avoid the exchange of dietary fat impacting on the intake of other nutrients (e.g. bioactive peptides, calcium and iodine) and potentially beneficial dairy components.

The plasma PL-FA profile associated with the high-SFA diet was characterised by higher proportions of pentadecanoic acid (C15:0) and vaccenic acid (C18:1 *n*−7 *trans*). These two FAs have been previously used as biomarkers of dairy fat consumption, as odd-chain SFAs and ruminant TFAs are synthesised in the rumen of cows before being integrated into the fat fraction of dairy foods [19, 38]. As plasma PL-FAs are thought to reflect short to medium-term dietary FA consumption [12, 16, 17], the importance of these two FAs in the high-SFA diet plasma PL-FA profile may be explained by a higher consumption of full-fat dairy products, which contributed to 39.6% of dietary total fat and 50.1% of dietary SFAs during the high-SFA diet. The strong association between dairy fat consumption and pentadecanoic acid or vaccenic acid in plasma PL from the RISSCI-1 study participants was further confirmed in prospective and cross-sectional multiple linear regression models, which identified these FAs as two independent predictors of dairy fat consumption among the 25 other FAs measured in plasma PL. In particular, the large effect estimates observed in linear regression models suggested that a large amount of dairy fat would need to be consumed to observe a 1% increase (%wt total FA) in the abundance of pentadecanoic or *trans*-vaccenic acid in plasma phospholipids. These findings from plasma PL-FAs are consistent with those from previous RCTs, which reported moderate but consistent associations between total dairy consumption and circulating levels of pentadecanoic acid in serum or plasma total lipids [50–52]. However, these findings from the RISSCI-1 study provide novel evidence for the utility of vaccenic acid as a biomarker for dairy fat consumption, a ruminant TFA that has been previously under studied in intervention studies [19]. The predictive accuracy of circulating pentadecanoic or vaccenic acids as biomarkers of dairy fat consumption, reflected by the predictive R^2^ value, was significantly improved when using prospective multiple regression models (i.e. changes between baseline and high-SFA diet) compared to cross-sectional models. This might provide an important area of future research for the use of these FAs in observational epidemiology studies, which often rely on a single measurement of dairy-specific FAs (e.g. pentadecanoic, heptadecanoic, or vaccenic acids) to investigate associations with mortality or incidence of cardiometabolic diseases [53–55].

In contrast to the high-SFA diet, the low-SFA diet was associated with higher abundances of long-chain MUFAs and n-6 PUFAs, such as eicosenoic and linoleic acids in plasma PL, which may reflect the increased dietary consumption of MUFAs and PUFAs from sunflower oil and vegetable spread during the low-SFA diet [16, 17]. Moreover, the low-SFA plasma PL-FA profile was also characterised by higher concentrations of long-chain SFAs (i.e. ranging from 18 to 22 carbons). These results might be partly explained by the endogenous synthesis of long-chain SFAs in humans together with the fat composition of sunflower oil, vegetable spreads, and nut-based snacks recommended during the low-SFA diet, which contain very small amounts of long-chain SFAs [56, 57]. In line with this hypothesis, a prospective study of changes in plasma PL-FA concentrations over 13 y among participants of the EPIC-Norfolk study reported that each additional 100 g/d of nut and seeds intake was associated with a 2.33% increase in plasma PL long-chain SFAs (20 to 24 carbons, 95% CI: 0.15–4.55) [58]. In addition, the low-SFA diet resulted in lower abundances of long-chain n-3 FAs in plasma PL. Since plasma PL-FA are expressed in relative (%wt) rather than absolute concentrations, the lower abundances of long-chain n-3 PUFAs in plasma PL after the low-SFA diet might represent higher abundances of other FAs. In particular, this may reflect the exchange of dietary SFA with mostly n-6 PUFA, in line with the consistent evidence on replacing dietary SFA with n-6 PUFA for cardiovascular disease (CVD) risk prevention [8].

A major strength of the RISSCI-1 dietary intervention was its success in replacing dietary SFAs with UFAs from commonly available commercial foods in healthy, free-living men living in the UK. The reduction of dietary SFAs achieved in the dietary intervention exceeded public health guidelines by reducing dietary SFA consumption to below 10%TE [8]. The dietary intervention was also reported to be well received by the participants, on the basis of self-reports and low attrition rate. This may be explained, in part, by the wide range of commercially available food products recommended and supplied during each dietary intervention period, which facilitated compliance, and minimised disruption to the participants’ habitual dietary habits.

Limitations of the dietary intervention included the use of self-reported dietary records, which may have influenced the eating behaviour of participants, and introduced bias towards healthier dietary patterns and under-reporting of energy intakes [59, 60]. Such self-reporting bias may partly account for the moderate proportion of under-reporting of energy intakes among participants at baseline (28%) and during the low-SFA diet (27%), which were similar to that observed in previous dietary intervention studies in free-living participants [11–13]. Interestingly, under-reporting of dietary energy was much less prevalent during the high-SFA diet (17%), which might, in part, be explained by increased awareness of the importance of accurate dietary records after being enrolled in the study. However, this might have been attenuated throughout the course of the 8-week intervention, as reflected in the higher degree of under-reporting observed at the end of the study, which may reflect participants’ fatigue. Moreover, dietary intakes were calculated using food composition databases, which could have introduced measurement errors through missing values and lack of diversity in food items. PUFAs (n-3 and n-6) were the main nutrients affected by this limitation, and their consumptions were estimated more accurately using the NDNS nutrient databank [21] to complement missing data from the CoFID database [23]. In addition, since food composition databases did not allow for the reliable estimation of the intake of specific FAs, dietary SFAs were considered as a whole. Although specific SFAs are known to exert different effects on markers of CVD risk, such as serum LDL-cholesterol [61], this was not of immediate relevance to the outcomes reported here. Another possible limitation of this study included the 4-week duration of each dietary intervention, which may not have been sufficient for plasma PL-FA concentrations to stabilise and potentially led to carry-over effects from the high- to the low-SFA diet. In particular, such carry-over effects may have underestimated the changes in plasma PL-FA abundances between the two diets. However, the observed changes in abundances of individual PL-FAs (Table 5) and patterns of PL-FAs (Fig. 2) both align with the dietary guidelines provided and were sufficient to reveal differences between the two dietary interventions. In addition, participants were healthy men, many with optimal BMI (between 18.5 and 24.9 kg/m^2^, *n* = 56, 52.8%), high self-reported physical activity levels (*n* = 52, 47.7%), and white ethnic background (*n* = 94, 86.2%), which may limit the generalisability of the study findings to a wider population. However, self-reported ethnicity from the RISSCI-1 closely match data from the 2011 Census in England and Wales [62]. Finally, the application of this food-exchange model in non-interventional ‘real-life’ settings may be affected by factors influencing food purchases, such as personal preference, financial and familial situations, as well as cultural background.

In conclusion, the RISSCI-1 dietary fat exchange model was successful in exchanging dietary SFAs for UFAs in healthy UK men, in accordance with current UK public health guidelines for adults. The replacement of dietary SFAs with UFAs, was based on commercially available foods and relied mostly on dairy foods, snacks, and cooking oil, and did not interfere with the overall dietary patterns of participants. Confirmation of the feasibility and efficacy of this food-based dietary exchange model will require its use in larger populations and intervention studies of longer duration.

## Supplementary Information

Below is the link to the electronic supplementary material.

Supplementary file1 (DOCX 25 kb)

## Data Availability

Not applicable.

## Abbreviations

%TE: % Total energy
AOAC: Association of analytical chemists
BMI: Body mass index
CVD: Cardiovascular disease
FA: Fatty acid
FAME: Fatty acid methyl ester
FID: Flame ionization detector
GC: Gas chromatograph
LDL-C: Low-density lipoprotein cholesterol
MUFAs: Monounsaturated fatty acids
NDNS: National Diet and Nutrition Survey
PL: Phospholipid
PUFAs: Polyunsaturated fatty acids
RCT: Randomised controlled trial
SACN: Scientific Advisory Committee on Nutrition
SD: Standard deviation
SFAs: Saturated fatty acids
TFAs: *trans* Fatty acids
UFAs: Unsaturated fatty acids
wt%: Weight %
