# Supplementary material for: Impact of a food-based dietary fat exchange model for replacing dietary saturated with unsaturated fatty acids in healthy men on plasma phospholipids fatty acid profiles and dietary patterns
Source: Eur J Nutr. 2022 Jun 6;61(7):3669–84. doi: 10.1007/s00394-022-02910-2 (PMC9464142; doi:10.1007/s00394-022-02910-2)
Supplement: Supplementary file 1 — Supplementary file1 (DOCX 25 kb) [file 394_2022_2910_MOESM1_ESM.docx]

**Supplementary table 1.** Definition of food categories used to assess dietary patterns in the RISSCI-1 study.

| **Food category** | **Example items** | **Calculation details** |
| --- | --- | --- |
| Fruits | Banana, apple, berries, etc. | Canned, stewed, and dried fruit as equivalent weight of whole fruit, including fruit within composite dishes |
| Whole vegetables | Cucumber, tomatoes, spinach, etc. | All cooked or raw vegetables, including tomato puree as equivalent weight of whole vegetable. |
| Pulses | Lentils, beans, chickpeas, etc. | Equivalent cooked weight |
| Vegetarian processed foods and ready meals | Potato dishes, pizza, salads, egg dishes, | Weight as consumed |
| Soups | any vegetable soup, including meat or fish soups | Weight as consumed |
| Cooking sauces | Tomato sauce, creamy sauces, pesto, etc. | Weight as consumed |
| Sauces and stock | Gravy, chicken stock, etc. | Weight as consumed |
| Nut butters | Peanut butter, Tahini paste, etc. | Weight as consumed |
| Nuts | Walnuts, hazelnuts, etc. | Weight as consumed |
| Seeds | Sesame seeds, etc. | Weight as consumed |
| Red and processed meats, offals | Beef, lamb, cured meats, sausages, etc. | Equivalent cooked weight, excluding waste (e.g. bones) |
| Poultry | Turkey, chicken, etc. | Equivalent cooked weight, excluding wastage (e.g. bones) |
| Meat alternatives | Quorn, tofu, etc. | Weight as consumed |
| Red and processed meat dishes | Meat pies, meat curry dishes, etc. | Weight as consumed |
| White fish | Cod, plaice, etc. | Equivalent cooked weight |
| Oily fish | Salmon, mackerel, etc. | Equivalent cooked weight |
| Shellfish | Mussels, clams, crab, etc. | Excluding wastage (e.g. shells) |
| Fish dishes | Fish pies, breaded fish, etc. | Oily and white fish, and shellfish included |
| Full-fat dairy foods | Whole milk, medium and full-fat cheese, full-fat yogurts, dairy desserts. | Weight as consumed |
| Reduced-fat dairy foods | Semi-skimmed and skimmed milk, low-fat and fat free yogurts, low-fat cheese | Weight as consumed |
| Dairy alternatives | Plant-based milks, plant-based yogurts, etc. | Included fortified and non-fortified dairy alternatives |
| Eggs | All types of eggs | Weight as consumed |
| Refined grains | Pasta, rice, etc. | Equivalent cooked weight |
| Refined grain foods | Bread, flour, crackers, etc. | Weight as consumed |
| Whole grains | Pasta, rice, etc. | Equivalent cooked weight |
| Whole grain foods | Bread, flour, crackers, etc. | Weight as consumed |
| Oats | Porridge and rolled oats | Equivalent dry weight |
| Condiments | Vinegar, mustard, salad dressing, herbs, spices, etc. | Weight as consumed |
| MUFA-rich fat | Olive oil, vegetable fat spread | Weight as consumed |
| PUFA-rich fat | Sunflower oil, vegetable fat spread | Weight as consumed |
| SFA-rich fat | Butter, animal fat, coconut fat | Weight as consumed |
| Biscuits and cakes | Sweet bakery products, biscuits, etc. | Weight as consumed |
| Savoury snacks | Crisps, crackers, corn/maize based snacks, etc. | Weight as consumed |
| Sugary products | Marmalades, jams, syrups, sugar, etc. | Weight as consumed |
| Sugar alternatives | Stevia, aspartame, etc. | Weight as consumed |
| Coffee | All coffee drinks | Weight as consumed |
| Tea | Green, black, herbal tea drinks | Weight as consumed |
| Sweetened drinks | Sodas, tonics, squashes, etc. | Equivalent ready to drink weight |
| Sugar free drinks | Sodas, tonics, squashes, etc. | Equivalent ready to drink weight |
| Alcoholic drinks | Beers, liqueurs, spirits, cocktails, etc. | Weight as consumed |

**Supplementary table 2.** Contribution of total dairy foods to nutrient intakes (%) in the RISSCI-1 study participants. ^a^

| **Nutrients** | **Baseline ^b^** | | **High-SFA diet ^c^** | | **Low-SFA diet ^d^** | |
| --- | --- | --- | --- | --- | --- | --- |
|  | **Mean, %** | **SD** | **Mean, %** | **SD** | **Mean, %** | **SD** |
| **Energy** | 10.6 | 6.4 | 20.8 | 6.3 | 6.3 | 3.0 |
| **Protein** | 14.0 | 8.5 | 20.9 | 7.8 | 15.3 | 6.9 |
| **Carbohydrates** | 5.4 | 4.2 | 5.9 | 3.5 | 6.6 | 3.6 |
| **Sugars** | 2.6 | 5.3 | 0.2 | 1.0 | 0.5 | 0.8 |
| **AOAC Fibre** | 0.6 | 1.7 | 0.1 | 0.7 | 0.4 | 1.1 |
| **Total fat** | 16.6 | 11.4 | 39.6 | 11.5 | 3.3 | 4.0 |
| **SFAs** | 28.5 | 17.5 | 50.1 | 12.6 | 8.1 | 8.4 |
| **MUFAs** | 12.5 | 9.6 | 35.8 | 12.5 | 2.5 | 3.3 |
| **PUFAs** | 4.2 | 4.9 | 13.7 | 7.4 | 0.4 | 0.9 |
| **n-3 PUFAs** | 5.7 | 7.9 | 19.7 | 13.8 | 0.4 | 1.5 |
| **n-6 PUFAs** | 2.9 | 3.7 | 10.6 | 7.3 | 0.2 | 0.7 |
| **TFAs** | 45.2 | 26.0 | 74.3 | 18.9 | 22.5 | 25.7 |
| **Cholesterol** | 21.7 | 16.5 | 44.0 | 19.4 | 13.0 | 16.4 |
| **Sodium** | 10.6 | 7.8 | 21.9 | 9.2 | 9.8 | 5.6 |
| **Iodine** | 50.5 | 22.4 | 66.1 | 18.5 | 61.7 | 20.9 |
| **Calcium** | 37.5 | 17.1 | 53.4 | 14.0 | 42.5 | 15.1 |

**Abbreviations: AOAC**, American Association of Analytical Chemists; **MUFAs**, monounsaturated fatty acids; **PUFAs,** polyunsaturated fatty acids; **SD** standard deviation; **SFAs**, saturated fatty acids; **TFAs**, trans fatty acids.

^a^ total dairy foods included milk, cheese, yogurt, dairy cream, butter, and dairy from milky drinks (e.g. milkshakes and cappuccino).

^b^ based on n=106 participants.

^c^ based on n=104 participants.

^d^ based on n=100 participants.
